# TLR7/8 activation induces autoimmune vasculopathy and causes severe pulmonary arterial hypertension

**DOI:** 10.1183/13993003.00204-2023

**Published:** 2023-07-20

**Authors:** Fu-Chiang Yeh, Chien-Nien Chen, Chong-Yang Xie, Nicoleta Baxan, Lin Zhao, Ali Ashek, Farah Sabrin, Allan Lawrie, Martin Wilkins, Lan Zhao

**Affiliations:** 1 National Heart and Lung Institute, Faculty of Medicine, Imperial College London, Hammersmith Hospital, London, UK; 2 Division of Rheumatology, Immunology and Allergy, Department of Internal Medicine, Tri-Service General Hospital, National Defense Medical Center, Taipei, Taiwan; 3 F-C. Yeh and C-N. Chen contributed equally as first authors

## Abstract

Growing evidence supports the contention that immune dysregulation and autoimmunity predispose and contribute to the pathological remodelling that characterise pulmonary arterial hypertension (PAH) [1, 2]. PAH is a common complication of connective tissue diseases (CTDs), especially systemic sclerosis (SSc) and systemic lupus erythematosus (SLE) [3]. In addition, idiopathic PAH (IPAH) patients exhibit immune cell infiltration of remodelled vessels, a shift in Th17/Treg axis and elevated levels of circulating cytokines and autoantibodies [2]. We have explored toll-like receptor 7 and 8 (TLR7/8)-induced autoimmunity in perpetuating vascular endothelial growth factor receptor antagonist Sugen 5416 (SU5416)-induced pulmonary endothelial injury and dysfunction in the initiation and further development of pulmonary vascular disease in rats (R-SU).

## Tweetable abstract

**This study introduced a combination of autoimmune stimuli (TLR-7/8 agonist) and endothelial injury (Sugen) and established a novel rat model representing immune–vascular pathological events mimicking human PAH associated with autoimmune disorders** https://bit.ly/3P3H5V0

*To the Editor:*

Growing evidence supports the contention that immune dysregulation and autoimmunity predispose and contribute to the pathological remodelling that characterise pulmonary arterial hypertension (PAH) [1, 2]. PAH is a common complication of connective tissue diseases (CTDs), especially systemic sclerosis (SSc) and systemic lupus erythematosus (SLE) [3]. In addition, idiopathic PAH (IPAH) patients exhibit immune cell infiltration of remodelled vessels, a shift in Th17/Treg axis and elevated levels of circulating cytokines and autoantibodies [2]. We have explored toll-like receptor 7 and 8 (TLR7/8)-induced autoimmunity in perpetuating vascular endothelial growth factor receptor antagonist Sugen 5416 (SU5416)-induced pulmonary endothelial injury and dysfunction in the initiation and further development of pulmonary vascular disease in rats (R-SU).

TLR7/8 are immune sentinels that selectively recognise subsets of RNA sequences crucial to antiviral responses. Human TLR7 is expressed in plasmacytoid dendritic cells (pDCs) and B cells, and produces type I interferon (IFN) signalling upon activation, while TLR8 in myeloid cells induces nuclear factor-κB-dependent cytokines [4]. Persistent TLR7/8 activation produces excessive pro-inflammatory signals which are often pathological; for example, enhanced TLR7 signalling triggers fatal cytokine storm in patients with viral pneumonia [5]. Abnormal TLR7-mediated IFN production by pDCs promotes autoreactive B cell expansion and repolarises T helper cells, which has been recognised as an autoimmunity signature [6]. Topical application of the TLR7/8 agonist resiquimod (R848) induces SLE-like autoimmune disorders in mice, resulting in elevated levels of cytokines, production of autoantibodies and multiple organ injuries; notably, these R848-induced autoimmune responses were eliminated in TLR7-deficient mice, suggesting that rodent TLR8 is less or non-functional [7]. SU5416 is well-recognised to induce endothelial cell apoptosis, a transient yet defining pathogenic event that underlies the development of pulmonary vascular remodelling [8]. SU5416 has been used in combination with other insults, such as chronic hypoxia, pneumonectomy, hypoxia-inducible factor-1α stimulation, vasoactive stressor and T cell depletion, to generate experimental pulmonary hypertension (PH) *in vivo* [8].

In this study, male Sprague–Dawley rats (180–200 g) were given a single subcutaneous injection of SU5416 (20 mg·kg^−1^), followed by topical application of TLR7/8 agonist R848 (R-SU) to the ears at 1.67 mg·kg^−1^ three times per week for 5 weeks (5W) with no exposure to hypoxia (figure 1a). At the initial stage, topical R848 caused erythematous skin changes on rat ears. Histology of R-SU rat ears at 5W showed epidermal hyperplasia and focal accumulation of CD103-positive cells at the epidermal–dermal junction (figure 1a). The 5W R-SU rats exhibited severe PH phenotypes, including significantly elevated mean pulmonary arterial pressure (PAP) (figure 1b) and right ventricle (RV) hypertrophy (figure 1c). Histological examination revealed obliterative vascular remodelling in 5W R-SU rat lungs (figure 1d). An additional group of rats (7W R-SU) was set up for longitudinal follow-up by cardiac magnetic resonance imaging (MRI) using a 9.4 T BioSpec scanner at 2- and 5-week timepoints, and an extended 7-week timepoint (maintained for further 2 weeks without R848 application). Notably, 7W R-SU rats maintained the elevated PAP and pulmonary vascular remodelling at a similar level as the 5W R-SU group (figure 1b and d), suggesting 5W to 7W as an ideal window to test pharmacological interventions. We observed no significant changes of the systemic pressure in either 5W or 7W R-SU groups. MRI data analysis confirmed a gradual declining RV performance along with PH progression (figure 1e) as evidenced by progressive RV hypertrophy and RV dilatation, as well as loss of ventriculo-arterial coupling and deteriorated RV function, *e.g.* declined RV ejection fraction (figure 1e).

**FIGURE 1:**
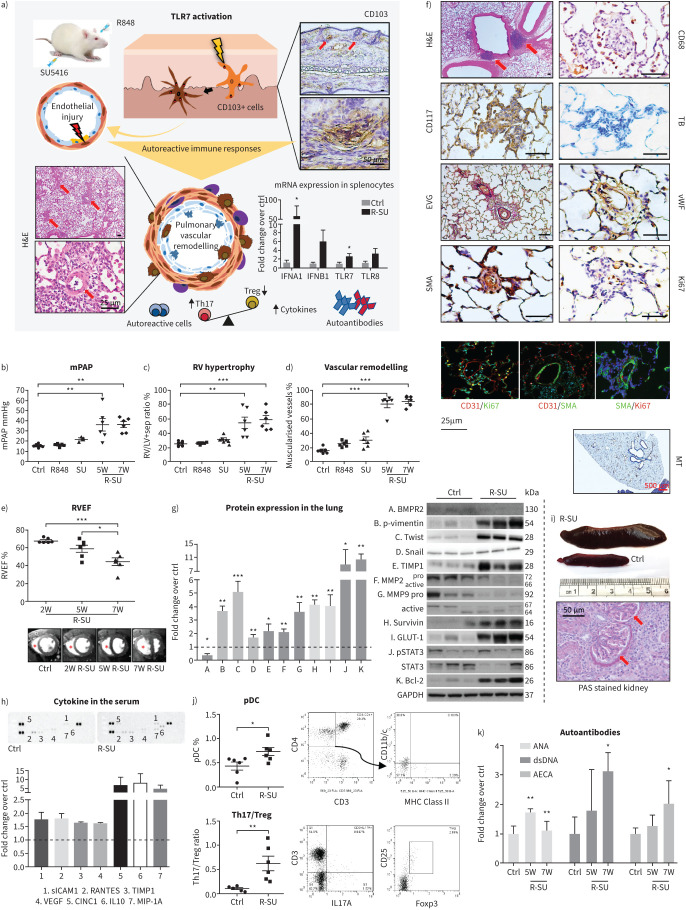
R848-SU5416 (R-SU) rat model of pulmonary hypertension. a) R-SU is established by combination of topical R848 application (1.67 mg·kg^−1^ three times per week for 5 weeks) on the rat ears and single subcutaneous injection of SU5416 (20 mg·kg^−1^). Immunohistochemical analysis demonstrated a focal accumulation of CD103^+^ cells at the epidermal–dermal junction of ear where R848 was applied (arrow); mRNA expressions of interferon (IFN) A1, IFNB1, toll-like receptor 7 (TLR7) and TLR8 were increased in the spleen (n=6), suggesting the involvement of plasmacytoid dendritic cells (pDCs) in response to TLR7/8 activation. The TLR7-driven autoimmunity perpetuates SU5416-induced injurious stimuli in the pulmonary vasculatures and eventually causes obliterative vascular remodelling. b) Mean pulmonary arterial pressure (mPAP); c) right ventricle (RV) hypertrophy calculated by Fulton's index as the weight ratio of RV to left ventricle (LV)+septum; d) percentages of muscularised pulmonary vessels in R-SU rats. Ctrl: healthy control; R848: R848 treated alone; SU: SU5416 treated alone; 5W: 5-week R848+SU5416 treated rats; 7W: 7-week R848+SU5416 treated rats. n=6. e) RV ejection fraction (RVEF) from different time points (2-week (2W), 5-week (5W), 7-week (7W)) of the same R-SU, measured by cardiac magnetic resonance imaging. n=6. f) Histological examination demonstrates infiltration of inflammatory cells and vascular remodeling in R-SU rat lungs. Haematoxylin and eosin (H&E) staining shows prominent bronchus-associated lymphoid tissues (arrows) around pulmonary vessels; CD68 staining macrophages; CD117 and Toluidine blue (TB) staining mast cells; Elastic Van Gieson (EVG) staining shows occlusive vascular lesions; von Willebrand factor (vWF) staining endothelial cells; smooth muscle actin (SMA) staining smooth muscle cells; Ki67 staining proliferating cells; double immunofluorescence staining with CD31/Ki67, CD31/SMA and SMA/Ki67; Masson's Trichrome (MT) staining connective tissues. g) Protein expression in R-SU rat lungs assessed by Western blotting. The data were generated from optical density measurements of individual bands normalised to GAPDH (pSTAT3 to total STAT3; active MMP2/9 to pro MMP2/9) and presented as fold change relative to control group. n=6. h) Proteom cytokine profiles in R-SU rat serum using R&D systems Panel A. Serum of six individual rats per group equally contributed to the pooled samples to incubate the profiler membranes. The experiment was duplicated with separate sample preparations. The data were generated from optical density measurements of individual dots and normalised to reference spots on each membrane, and presented as fold change relative to control group. i) Enlarged spleen from R-SU rats compared with controls, the quantification was accessed by the weight ratio of spleen over body weight; Ctrl: 2.04±0.22×10^−3^ *versus* R-SU: 8.50±1.31×10^−3^ (p<0.001). Periodic acid Schiff (PAS) stained kidney sections from R-SU rats reveal glomerulonephritis. j) Fluorescence-activated cell sorting analysis of peripheral mononuclear cells from R-SU rats shows increased frequencies of CD3^−^CD4^+^CD11b/c^low^MHC class II^+^ cells, referring activated pDCs, and an increased ratio of Th17 (defined by CD3^+^CD4^+^IL17A^+^) to Treg cells (defined by CD3^+^CD4^+^CD25^+^Foxp3^+^). n=6. k) Plasma levels of autoantibodies: anti-nuclear antibody (ANA), anti-double stranded DNA antibody (anti-dsDNA) and anti-endothelial cell antibody (AECA) in R-SU rats detected by ELISA. Ctrl: healthy control; 5W: 5-week R-SU rats; 7W: 7-week R-SU rats. n=6. All values are presented as mean±sem. Differences were assessed by one-way ANOVA (multiple groups) or unpaired t-test (two groups). *: p<0.05; **: p<0.01; ***: p<0.001. Primers used in qRT-PCR: IFNA1 (F-CAGCAGATCCAGAAGDCTCAARC;R-CAGGCACAGGGRCTGTGTTT), IFNB1 (F-TGCTGTGCTTCTCCACCACT;R-TAGTCTCATTCCACCCAGTGCT), TLR7 (F-TTTCCCAGAGCATACAGCTCAG;R- CACTCAAGGACAGAACTGCTGC), TLR8 (F:CCAGAGTCTTCCAAACTTGGCAAC;R-CAAGGCCTTGCCATAAGCAGTACA).

Histological examination of the R-SU rat lungs showed extensive perivascular immune cell recruitment/infiltration in the lungs. Neither R848 nor SU5416 alone was sufficient to cause evident PH pathology. The R848 and SU5416 “double hit” R-SU rat exhibited conspicuous inflammatory cell infiltrates around the obliterative vascular lesions in the lungs, as well as in enlarged bronchus-associated lymphoid tissues (BALTs) (figure 1f). Previous studies from human IPAH and animal models of PH induced by hypoxia and monocrotaline have shown that BALTs were a source of pathological autoantibodies, *e.g.* anti-endothelial cell antibodies (AECAs), that cause endothelial apoptosis [9]. In R-SU rat lungs, immunohistochemistry (figure 1f) demonstrated accumulation of mast cells (CD117 along with toluidine blue stained) and CD68^+^ macrophages surrounding the remodelled vessels, and a higher presence of Ki67 positive proliferating vascular cells including both endothelial (von Willebrand factor, vWF) and smooth muscle cells (smooth muscle actin, SMA), respectively. In addition, there was excessive formation of fibrotic connective tissues deposition (Masson's trichrome blue staining; figure 1f) in the lungs, indicating lung tissue damage and inflammation in R-SU rats. This resembles the distinctive pathology observed in severe human PAH, where immune cell infiltrates co-reside with proliferating vascular cells in concentric neointimal lesions. Consistently, R-SU rats presented typical features of PH-related endothelial dysfunction, including bone morphogenetic protein receptor type 2 (BMPR2) deficiency, higher signal transducer and activator of transcription 3 (STAT3) phosphorylation and phospho-vimentin (endothelial-to-mesenchymal transition (EndoMT)) [10] in the lungs (figure 1g). In addition, anti-apoptotic markers such as survivin and Bcl-2 were highly expressed in R-SU rat lungs together with the elevated GLUT1 expression reflecting glycolytic shifts seen with the pro-proliferative phenotypes (figure 1g). Interestingly, R-SU rats demonstrated a prominent elevation of cytokines/chemokines including RANTES (regulated on activation, normal T cell expressed and secreted), interleukin (IL)-10, soluble intercellular adhesion molecule (sICAM) and macrophage inflammatory protein-1A. RANTES expression indicates the integrity of downstream signals in response to IFN production (figure 1h). In line with increased matrix proteins (MMP2/MMP9/TIMP1) expression in R-SU rat lungs, RANTES elevation suggests persistent chemotactic responses and ongoing remodelling of pulmonary extracellular matrix which facilitate immune cell extravasation, migration and infiltration.

R-SU rats developed lupus-like systemic autoimmune responses with multiple organ involvements, such as splenomegaly (>4 fold-change in the ratio of spleen to body weight) and glomerulonephritis (figure 1i) [7]. Type I IFN (IFNA1/IFNAB1) and TLR7/8 expression were highly increased in R-SU rat splenocytes, proving the responses to R848. Importantly, R-SU rats exhibited an increased cell frequency of circulating CD3^−^CD4^+^CD11b/c^low^MHC class II^+^ subset, known homologues to human pDCs [11], as well as an increased Th17 (CD3^+^CD4^+^IL17A^+^)/Treg (CD3^+^CD4^+^CD25^+^Foxp3^+^) ratio in peripheral blood cells (figure 1j). Repolarisation towards Th17 cells upon TLR-7 activation and reduction of regulatory T cells (Tregs) signify loss of self-tolerance and increased autoimmunity, which has been described in correlation with disease severity and prognosis in various forms of human PAH as well as autoimmune disorders [12, 13]. Furthermore, plasma levels of autoantibodies were markedly increased in R-SU rats, including AECAs, antinuclear and anti-dsDNA antibodies (figure 1k). Production of autoantibodies is also a common immunopathy of PAH and autoimmune disorders. It is worth noting that the TLR7 agonist leads to a fatal lupus autoimmunity involving systemic hypertension and vascular inflammation in female mice [14]; we suspect the higher predisposition to TLR7-driven autoimmune responses in females might be attributed to the gene–dose effect of biallelic X-chromosome expression [15]. For this reason, adult male rats were chosen for this study and no significant changes in systemic circulation were noted in R-SU rats.

This study highlights the complex immune–vascular interactions involved in pulmonary vascular diseases associated with autoimmune/infectious disorders. Compared with existing PH experimental models, such as the Sugen-hypoxia rats, R-SU rats display prominent inflammatory cells accumulation in the perivascular regions of the remodelled vessels in the lungs. R-SU rats manifest the immune–vascular pathological events representative of human PAH and other relevant pulmonary diseases (*e.g.* pulmonary fibrosis, viral pneumonia due to COVID-19), and serve as a novel and important experimental tool in the pulmonary vascular research field.

## Shareable PDF

10.1183/13993003.00204-2023.Shareable1 This one-page PDF can be shared freely online.
Shareable PDF ERJ-00204-2023.Shareable
