# Supplementary material for: TLR7/8 activation induces autoimmune vasculopathy and causes severe pulmonary arterial hypertension
Source: Eur Respir J. 2023 Jul 20;62(1):2300204. doi: 10.1183/13993003.00204-2023 (PMC10356963; doi:10.1183/13993003.00204-2023)
Supplement: Supplementary file 1 [file ERJ-00204-2023.Shareable.pdf]

EUROPEAN RESPIRATORY JOURNAL
RESEARCH LETTER
F-C. YEH ET AL.

SHAREABLE PDF

TLR7/8 activation induces autoimmune vasculopathy and causes severe pulmonary arterial hypertension

Fu-Chiang Yeh[1,2,3], Chien-Nien Chen[1,3], Chong-Yang Xie[1], Nicoleta Baxan[1], Lin Zhao [1], Ali Ashek[1], Farah Sabrin[1], Allan Lawrie [1], Martin Wilkins [1] and Lan Zhao [1]

[1]National Heart and Lung Institute, Faculty of Medicine, Imperial College London, Hammersmith Hospital, London, UK. [2]Division of Rheumatology, Immunology and Allergy, Department of Internal Medicine, Tri-Service General Hospital, National Defense Medical Center, Taipei, Taiwan. [3]F-C. Yeh and C-N. Chen contributed equally as first authors.

Corresponding author: Lan Zhao (l.zhao@imperial.ac.uk)

Check for updates

Shareable abstract (@ERSpublications)
This study introduced a combination of autoimmune stimuli (TLR-7/8 agonist) and endothelial injury (Sugen) and established a novel rat model representing immune–vascular pathological events mimicking human PAH associated with autoimmune disorders https://bit.ly/3P3H5V0

Cite this article as: Yeh F-C, Chen C-N, Xie C-Y, *et al*. TLR7/8 activation induces autoimmune vasculopathy and causes severe pulmonary arterial hypertension. *Eur Respir J* 2023; 62: 2300204 [DOI: 10.1183/13993003.00204-2023].

This single-page version can be shared freely online.

Copyright ©The authors 2023.

This version is distributed under the terms of the Creative Commons Attribution Licence 4.0.

Received: 3 Feb 2023
Accepted: 30 May 2023

To the Editor:

Growing evidence supports the contention that immune dysregulation and autoimmunity predispose and contribute to the pathological remodelling that characterise pulmonary arterial hypertension (PAH) [1, 2]. PAH is a common complication of connective tissue diseases (CTDs), especially systemic sclerosis (SSc) and systemic lupus erythematosus (SLE) [3]. In addition, idiopathic PAH (IPAH) patients exhibit immune cell infiltration of remodelled vessels, a shift in Th17/Treg axis and elevated levels of circulating cytokines and autoantibodies [2]. We have explored toll-like receptor 7 and 8 (TLR7/8)-induced autoimmunity in perpetuating vascular endothelial growth factor receptor antagonist Sugen 5416 (SU5416)-induced pulmonary endothelial injury and dysfunction in the initiation and further development of pulmonary vascular disease in rats (R-SU).
